# Effect of coffee consumption on thyroid function: NHANES 2007-2012 and Mendelian randomization

**DOI:** 10.3389/fendo.2023.1188547

**Published:** 2023-06-07

**Authors:** Guoxu Zhao, Zhao Wang, Jinli Ji, Rongjun Cui

**Affiliations:** ^1^ Mudanjiang Medical University, Mudanjiang, China; ^2^ Chungnam National University School of Medicine, Daejeon Gwangyeoksi, Republic of Korea

**Keywords:** coffee, thyroid function, NHANES, Mendelian randomization, machine learning

## Abstract

**Background:**

Coffee is one of the most consumed beverages worldwide, but the effects on the thyroid are unknown. This study aims to examine the association between coffee and thyroid function.

**Methods:**

Participant data (≥ 20 years, n = 6578) for the observational study were obtained from NHANES 2007-2012. Analysis was performed using weighted linear regression models and multiple logistic regression models. Genetic datasets for Hyperthyroidism and Hypothyroidism were obtained from the IEU database and contained 462,933 European samples. Mendelian randomization (MR) was used for the analysis, inverse variance weighting (IVW) was the main method of analysis.

**Results:**

In the model adjusted for other covariates, participants who drank 2-4 cups of coffee per day had significantly lower TSH concentrations compared to non-coffee drinkers (b=-0.23, 95% CI: -0.30, -0.16), but no statistically significant changes in TT4, FT4, TT3 and FT3. In addition, participants who drank <2 cups of coffee per day showed a low risk of developing subclinical hypothyroidism. (OR=0.60, 95% CI: 0.41, 0.88) Observational studies and MR studies have demonstrated both that coffee consumption has no effect on the risk of hyperthyroidism and hypothyroidism.

**Conclusions:**

Our study showed that drinking <2 cups of coffee per day reduced the risk of subclinical hypothyroidism and drinking 2-4 cups of coffee reduced serum TSH concentrations. In addition, coffee consumption was not associated with the risk of hyperthyroidism and hypothyroidism.

## 1 Introduction

Coffee is among the most consumed beverages worldwide, with approximately 2.25 billion cups consumed daily, amounting to around 500 billion cups per year, as reported by the National Coffee Association (1). It is estimated that over 1,000 compounds can be found in coffee, with the most common being caffeine, chlorogenic acid, and melanoidins. The health effects of coffee consumption have piqued academic interest for many years. Numerous studies have demonstrated that coffee consumption is linked to a reduced risk of various chronic diseases (including type 2 diabetes and cardiovascular disease), cancer, and neurodegenerative diseases such as Parkinson’s disease (2). However, the impact on thyroid function remains unclear. The thyroid is the largest endocrine gland in the body (3). Thyroid hormone (TH) is synthesized and secreted by follicular epithelial cells, regulated by thyroid-stimulating hormone (TSH), and stored in the follicular compartment in colloidal form. TH broadly regulates growth, development, metabolism, and other bodily functions (4, 5). Thyroid hormones are iodides of tyrosine and primarily consist of triiodothyronine (T3) and thyroxine (T4). The active free thyroid hormones in the body include free triiodothyronine (FT3) and free thyroxine (FT4) (6). When FT3 and FT4 are depleted in the body, total T3 (TT3) and total T4 (TT4) are converted to FT3 and FT4, which continue to function as thyroid hormones (7, 8). Various lifestyle habits have been shown to cause changes in thyroid hormone levels, such as smoking, alcohol consumption, diet, and exercise (9). In previous animal experiments, caffeine, a substance found in coffee, inhibited TSH secretion by releasing hypothalamic growth inhibitory hormone after intraperitoneal injection into rats (10). However, no study on the effects on the thyroid gland after long-term coffee intake has been conducted. The National Health and Nutrition Examination Survey (NHANES) is a biennial survey of the U.S. population that employs a multi-stage probability sampling design, combining interviews, questionnaires, physical examinations, and laboratory data to assess the health and nutritional status of the population (11). Mendelian randomization (MR) analysis is widely used for causal inference in epidemiology, with the core concept being the use of genetic variation as an instrument variable (IV) to model and test the causal relationship between exposure factors and disease (12). The methodology of the MR study is akin to that of a randomized controlled trial (RCT) because parental alleles are randomly assigned to offspring according to Mendel’s law during gamete formation, making the MR study equivalent to a naturally occurring RCT in a population (13). Simultaneously, genetic variants are formed before birth and persist throughout life, allowing MR studies to effectively avoid the influence of reverse causality (14). Therefore, in our study, we will employ a combination of NHANES and MR analysis in an observational study to explore whether coffee consumption causes changes in serum TT4, TT3, FT4, FT3, and TSH concentrations and the causal effect of coffee consumption on thyroid disorders, including hyperthyroidism and hypothyroidism.

## 2 Materials and methods

### 2.1 Study samples in NHANES

**Figure 1:**
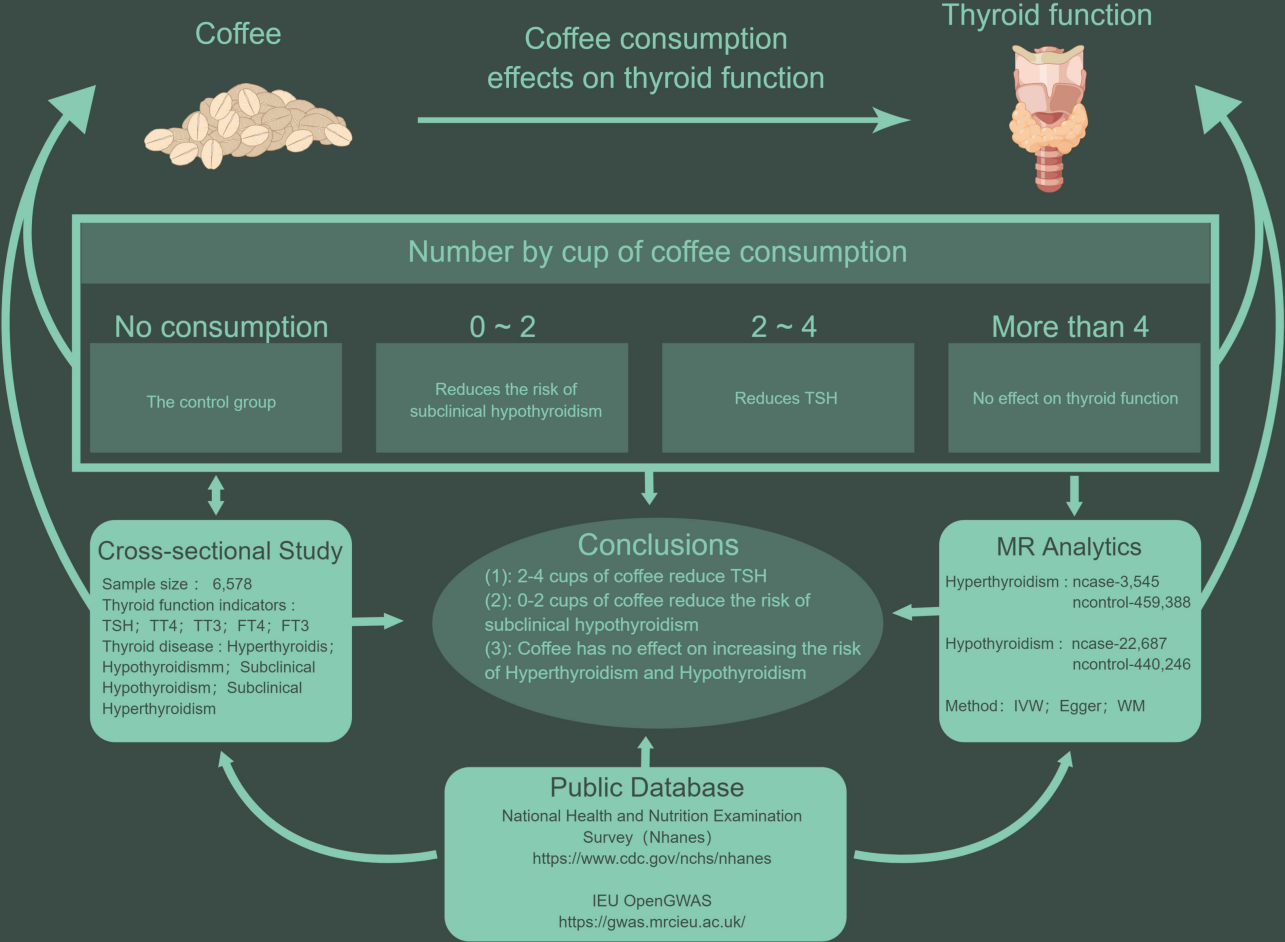
The line of research for this study.

The full process of this study is shown in **Figure 1**. Our study selected data on sub-jects from 2007-2012, a time interval chosen because it was the only time interval during which data on thyroid function were collected by NHANES. A total of 30,442 participants from NHANES 2007-2012, subjects aged 20 years and older were enrolled in our study. In addition, among all subjects, we excluded the following individuals: (1) Individuals with missing thyroid function test indicators. (2) Individuals lacking coffee consumption data. (3) Individuals with incomplete data on education status, diabetes, hypertension, BMI, hyperlipidemia, alcohol consumption, and smoking status. Final study sample size n=6,578 (weighted n = 63,453,885).

### 2.2 Coffee consumption data collection

Food frequency questionnaires and two 24-hour dietary recalls collected during NHANES 2007-2012 were used to obtain coffee consumption data. As presented in previous studies, coffee consumption in NHANES was correlated with specific 8-digit food codes (codes beginning with 921) in the Food and Nutrition Database for Dietary Studies (FNDDS) (15). We selected participants who had two 24-hour dietary recalls and used the average coffee consumption for the two 24-hour recalls. In this study the size of a cup of coffee was defined as 283.5 grams (16). In addition, the coffee intake was further divided into four groups: 0, <2, 2-4, and >4 cups/day.

### 2.3 Thyroid function assessment

FT3, TT3, FT4, TT4 and TSH data in the study were obtained from NHANES laboratory data. A competitive binding immunoenzymatically assay was employed for T3, FT3, and T4 measurements, while a two-step enzyme immunoassay was utilized for FT4 assessment. The evaluation of TSH was carried out using the Access HYPER sensitive human thyroid-stimulating hormone (hTSH) assay, which is a 3rd generation two-site immunoenzymatically assay (17, 18). Diagnosis of hyperthyroidism based on TSH<0.45mIU/and FT4>1.6ng/dL, and hypothyroidism based on TSH>4.5mIU/L and FT4<0.6ng/dL. Sub-clinical hyperthyroidism was diagnosed according to TSH<0.45mIU/L and 0.6<FT4<1.6ng/dL, and subclinical hypothyroidism was diagnosed according to TSH>4.5mIU/L and 0.6<FT4<1.6ng/dL (19).

### 2.4 Acquisition of covariates

Covariates in the study included age, gender (Male and Female), ethnicity (White, Black, Mexican and Other), education (High School Grad/GED or Equivalent, less than 9th Grade, 9th-11th Grade, College graduate or above), body mass index (BMI), smoking (Never, Former, Now), and alcohol use (Never, Former, Mild, Moderate, Heavy). In addition, by screening related studies, we additionally included urine iodine, diabetes (No and Yes), hypertension (No and Yes) and hyperlipidemia (No and Yes) data for analysis (20–24).

### 2.5 Acquisition of IV of MR

The IV used in this study was obtained from a study by Li et al. (23). We used two groups of IV for MR analysis. The first group IV (IV1) uses the number of cups of coffee consumed per day (cups/day) by coffee consumers as an instrument, and the second group IV (IV2) compares regular versus infrequent coffee consumers. After screening for P<5×10^-8^ and excluding those SNPs in a state of linkage disequilibrium (r2 = 0.01, 10,000 kb), 6 and 3 SNPs were finally included in IV1 and IV2, respectively.

### 2.6 Genetic data of hyperthyroidism and hypothyroidism

Genetic data for hyperthyroidism were obtained from a study compiled and published by Ben Elsworth et al. in 2018, contains 3545 European cases and 459,388 European controls, including a total of 9,851,867 SNPs. Genetic data for hypothyroidism were also obtained from the Ben Elsworth study, which included 22,687 European cases and 440,246 European controls. Genetic data for hyperthyroidism and hypothyroid-ism can be obtained from https://gwas.mrcieu.ac.uk/datasets/ukb-b-20289/ and https://gwas.mrcieu.ac.uk/datasets/ukb-b-19732/.

### 2.7 Statistical analysis

All statistical analyses were performed in R software (4.2.1), and survey data in **Table 1** were summarized by descriptive statistics, one-way ANOVA for continuous variables, and chi-square tests for categorical variables to assess the association be-tween coffee consumption and other factors in different groups. We used the sampling weights provided by NHANES for weighting (21). Weighted generalized linear regression models were used to assess the relationship between adjusted coffee consumption and serum thyroid function indicators. In addition, we used weighted multivariate adjusted logistic regression to calculate the odds ratio (OR) and 95% confidence interval (CI) for thyroid disease in the different coffee consumption groups. All analyses considered the NHANES complex multi-stage sampling design and p < 0.05 was considered statistically significant. The MR analyses were all performed using the TwoSampleMR package (13). In this study, multiple SNPs were used as IVs for the MR study. The association of individual SNPs was first performed, and the Wald ratio was calculated for each SNP, and then the Wald ratios were combined using the inverse variance weighted (IVW) method to assess the association between coffee consumption and thyroid disease. To check the robustness of the results, we additionally used MR-Egger regression and weighted median estimator (WME) for additional analysis, these three calculation methods have different validity assumptions for IV. In addition, the mean pleiotropic effect of genetic variants could be assessed using the intercept of the MR-Egger regression (tested using P < 0.05) and the Cochran’s Q test was used to determine the heterogeneity between the causal estimates of different genetic variants. Eventually a sensitivity analysis was also performed using the leave-one-out method.

**Table 1:** Baseline table of participants categorized by number of cups of coffee consumed.

Variable	Total	0	<2	2-4	>4	P
N(Unweighted)	6578(100.00)	2741(41.66)	1969(29.93)	1204(18.30)	664(10.09)	
Age	51.05(50.28,51.82)	46.36(45.15,47.57)	52.84(51.74,53.94)	56.19(54.96,57.43)	55.24(53.73,56.75)	0.01
Sex						0.01
Male	3248(49.38)	1358(49.40)	876(43.86)	618(52.73)	396(59.71)	
Female	3330(50.62)	1383(50.60)	1093(56.14)	586(47.27)	268(40.29)	
Eth						0.01
white	3201(48.66)	1217(42.71)	736(35.73)	721(60.01)	527(80.31)	
Black	1298(19.73)	779(29.83)	345(16.44)	142(10.32)	32(4.99)	
Mexican	1010(15.35)	391(14.07)	417(23.49)	159(14.79)	43(5.34)	
Other	1069(16.25)	354(13.39)	471(24.34)	182(14.88)	62(9.36)	
Edu						0.01
Less than 9th Grade	723(10.99)	230(8.62)	285(15.77)	151(14.40)	57(8.63)	
9-11th Grade	1061(16.13)	440(15.63)	347(17.74)	163(13.87)	111(16.71)	
High School Grad/GED or Equivalent	1562(23.75)	671(24.50)	434(22.85)	287(24.56)	170(26.78)	
College graduate or above	3232(49.13)	1400(51.25)	903(43.64)	603(47.17)	326(47.87)	
Smoke						0.01
Never	3492(53.09)	1734(64.81)	1112(56.56)	471(40.84)	175(26.82)	
Former	1745(26.53)	533(18.60)	528(25.94)	456(36.98)	228(36.72)	
Now	1341(20.39)	474(16.59)	329(17.50)	277(22.18)	261(36.46)	
Alcohol User						0.01
Never	931(14.15)	475(17.72)	329(18.55)	91(8.66)	36(4.77)	
Former	1299(19.75)	504(17.55)	375(18.33)	262(23.84)	158(23.84)	
Mild	2081(31.64)	761(27.37)	628(29.29)	465(36.93)	227(37.90)	
Moderate	972(14.78)	406(14.87)	275(14.21)	177(13.29)	114(15.61)	
Heavy	1295(19.69)	595(22.48)	362(19.63)	209(17.28)	129(17.88)	
DM						0.01
No	5686(86.44)	2433(88.57)	1669(84.28)	1020(84.30)	564(86.14)	
Yes	892(13.56)	308(11.43)	300(15.72)	184(15.70)	100(13.86)	
Hyperlipidemia						0.01
No	1617(24.58)	797(28.38)	428(20.86)	255(22.45)	137(22.32)	
Yes	4961(75.42)	1944(71.62)	1541(79.14)	949(77.55)	527(77.68)	
Hypertension						0.09
No	3727(56.66)	1642(58.61)	1088(53.22)	637(54.87)	360(55.61)	
Yes	2851(43.34)	1099(41.39)	881(46.78)	567(45.13)	304(44.39)	
Hyperthyroidism						0.33
No	6562(99.76)	2734(99.72)	1965(99.88)	1202(99.86)	661(99.42)	
Yes	16(0.24)	7(0.28)	4(0.12)	2(0.14)	3(0.58)	
Hypothyroidism						0.17
No	6544(99.48)	2728(99.68)	1961(99.71)	1197(99.78)	658(99.11)	
Yes	34(0.52)	13(0.32)	8(0.29)	7(0.22)	6(0.89)	
Subclinical Hyperthyroidism						0.96
No	6373(96.88)	2661(96.80)	1905(96.81)	1162(96.41)	645(96.63)	
Yes	205(3.12)	80(3.20)	64(3.19)	42(3.59)	19(3.37)	
Subclinical Hypothyroidism						0.24
No	6339(96.37)	2637(96.19)	1905(97.27)	1157(95.31)	640(96.45)	
Yes	239(3.63)	104(3.81)	64(2.73)	47(4.69)	24(3.55)	
BMI (kg/m ^2^)	29.08(28.80,29.35)	29.36(28.96,29.76)	28.70(28.35,29.04)	29.23(28.71,29.76)	28.74(28.17,29.32)	0.02
TT3 (ng/dL)	112.20(110.97,113.44)	113.90(112.17,115.62)	111.55(110.08,113.02)	110.59(108.15,113.03)	110.22(108.22,112.22)	0.01
TT4 (ug/dL)	7.93(7.85,8.00)	7.93(7.84,8.03)	7.99(7.89,8.10)	7.89(7.76,8.02)	7.78(7.63,7.94)	0.03
FT3 (pg/mL)	3.15(3.13,3.17)	3.18(3.15,3.21)	3.13(3.10,3.16)	3.13(3.09,3.17)	3.10(3.06,3.15)	0.02
FT4 (ng/dL)	0.80(0.79,0.81)	0.80(0.79,0.81)	0.80(0.79,0.81)	0.80(0.79,0.82)	0.80(0.78,0.82)	0.98
TSH (mIU/L)	2.01(1.90,2.12)	1.97(1.86,2.08)	2.02(1.86,2.17)	1.95(1.83,2.07)	2.30(1.79,2.81)	0.52
Urinary Iodine	285.45(242.56,328.34)	302.00(229.73,374.28)	297.34(205.32,389.35)	277.98(205.63,350.33)	191.66(163.06,220.25)	0.01

Edu, education, Eth, ethnicity Categorical variables show percentages, continuous variables show means and confidence intervals. A cup of coffee cup is defined as 283.5 grams.

## 3 Results

### 3.1 Baseline characteristics of participants

After screening as required, a total of 6578 subjects were included in this study, and among the general demographic data, 3330 (50.62%) were female, 3201 (48.66%) were white, 3232 (49.13%) were College graduate or above, 3492 (53.9%) were non-smokers, and 5647 (85.8%) were alcohol users above their corresponding groups. In addition, a total of 4961 cases were diagnosed with Hyperlipidemia, 2851 with Hyper-tension, and 892 with DM. 494 of these subjects were diagnosed with thyroid abnormalities, including 16 patients with hyperthyroidism, 34 with hypothyroidism, 205 with subclinical hyperthyroidism, and 239 with subclinical hypothyroidism (**Table 1**).

### 3.2 Relationship between coffee consumption and serum thyroid function indicators

In our study, 3837 (58.3%) were coffee drinkers, with <2 cups accounting for 29.93% of participants. After adjusting for gender, education, race, age, smoking, alcohol consumption, diabetes, hyperlipidemia, hypertension, BMI and urinary iodine, we found that those who consumed 2-4 cups of coffee per day had significantly lower levels of TSH compared to those who did not drink coffee. (b=-0.23, 95% CI: -0.30, -0.16), but there was no statistically significant association for TT4, FT4, TT3 and FT3 (p>0.05) (**Table 2**).

**Table 2:** Effect of coffee consumption on serum thyroid function indicators.

Variable	Coffee	Beta	SE	t value	P
TSH	ref	ref	ref	ref	ref
	<2	-0.06	0.09	-0.62	0.54
	2-4	-0.23	0.07	-3.27	0.003 **
	>4	0.11	0.26	0.44	0.66
TT4	ref	ref	ref	ref	ref
	<2	-0.06	0.07	-0.77	0.45
	2-4	-0.07	0.08	-0.81	0.43
	>4	-0.06	0.10	-0.59	0.56
TT3	ref	ref	ref	ref	ref
	<2	-0.43	0.91	-0.47	0.64
	2-4	0.02	1.28	0.02	0.99
	>4	-0.67	1.25	-0.53	0.60
FT4	ref	ref	ref	ref	ref
	<2	-0.01	0.01	-1.44	0.16
	2-4	-0.01	0.01	-1.00	0.32
	>4	-0.01	0.01	-0.69	0.49
FT3	ref	ref	ref	ref	ref
	<2	-0.01	0.02	-0.60	0.56
	2-4	0.02	0.03	0.68	0.50
	>4	-0.02	0.02	-1.03	0.31

**Tips less than 0.05, **Tips less than 0.01, Models adjusted for age, sex, education, ethnicity, smoking, alcohol, DM, hypertension, hyperlipidemia, BMI, and urinary iodine concentration.

### 3.3 Relationship between coffee consumption and thyroid disorders

Weighted logistic regression results showed that consumption of 0-2 cups of coffee per day was negatively associated with the risk of developing subclinical hypothyroid-ism. (OR=0.60, 95% CI: 0.41, 0.88), but there was no significant association between coffee intake and thyroid disease in other coffee consumption categories (**Table 3**).

**Table 3:** Effect of coffee consumption on serum thyroid function indicators.

Variable	Coffee	OR	95%CI		P
Hyperthyroidism	ref	ref	ref	ref	ref
	<2	0.33	0.05	2.20	0.26
	2-4	0.41	0.04	4.03	0.45
	>4	1.69	0.16	17.59	0.66
Hypothyroidism	ref	ref	ref	ref	ref
	<2	0.94	0.26	3.36	0.92
	2-4	0.54	0.13	2.24	0.40
	>4	1.98	0.52	7.45	0.32
Subclinical. Hyperthyroidism	ref	ref	ref	ref	ref
	<2	0.93	0.61	1.40	0.72
	2-4	1.03	0.58	1.82	0.93
	>4	0.98	0.46	2.07	0.96
Subclinical. Hypothyroidism	ref	ref	ref	ref	ref
	<2	0.60	0.41	0.89	0.015*
	2-4	0.89	0.59	1.36	0.61
	>4	0.65	0.25	1.72	0.40

**Tips less than 0.05, **Tips less than 0.01, Models adjusted for age, sex, education, ethnicity, smoking, alcohol, DM, hypertension, hyperlipidemia, BMI, and urinary iodine concentration.

### 3.4 MR analysis

The results of the study showed that coffee consumption calculated by IVW, WME and MR-Egger regression methods for IV1 and IV2 were not statistically significant with hyperthyroidism or hypothyroidism (**Table 4**), implying that there was no causal relationship between coffee consumption on hyperthyroidism and hypothyroidism in the population. We then validated the reliability of our results. The results showed that there was no heterogeneity or pleiotropy in our study. (S1 and S2) (p>0.05). Sensitivity analysis of the IVW results using the leave-one-out method showed that the elimination of SNPs one by one did not reveal that a particular SNP caused a significant change in the results, and no SNPs with a strong effect on the results were found in IV, indicating that the effect ORs derived from the previous IVW method were more robust.

**Table 4:** OR estimates and 95% CI for IVW, WME and MR-Egger regression.

IV	Exposure	Outcome	Method	nSNP	OR	95%CI		P
IV_1	Coffee	Hyperthyroidism	MR Egger	6	1.00	1.00	1.01	0.16
	Coffee	Hyperthyroidism	Weighted median	6	1.00	1.00	1.00	0.24
	Coffee	Hyperthyroidism	IVW	6	1.00	1.00	1.00	0.17
IV_2	Coffee	Hyperthyroidism	MR Egger	3	1.00	1.00	1.01	0.50
	Coffee	Hyperthyroidism	Weighted median	3	1.00	1.00	1.00	0.24
	Coffee	Hyperthyroidism	IVW	3	1.00	1.00	1.00	0.21
IV_1	Coffee	Hypothyroidism	MR Egger	6	1.01	0.99	1.02	0.28
	Coffee	Hypothyroidism	Weighted median	6	1.01	1.00	1.01	0.06
	Coffee	Hypothyroidism	IVW	6	1.00	1.00	1.01	0.21
IV_2	Coffee	Hypothyroidism	MR Egger	3	1.01	1.00	1.02	0.24
	Coffee	Hypothyroidism	Weighted median	3	1.00	1.00	1.01	0.06
	Coffee	Hypothyroidism	IVW	3	1.00	1.00	1.01	0.29

IVW, Inverse variance weighting.

## 4 Discussion

Coffee consumption ranks second only to water, yet the debate over its benefits and risks continues (25). Epidemiological studies investigating the relationship between coffee consumption and thyroid function are scarce. Hyperthyroidism is a condition resulting from excessive production of thyroid hormones. Common symptoms include weight loss, rapid heart rate, anxiety, and irritability. Serious complications may involve thyroid crisis, atrial fibrillation, and bone fractures. On the other hand, hypothyroidism is characterized by a deficiency in thyroid hormones, potentially leading to symptoms such as weight gain, fatigue, and depression. Severe complications of hypothyroidism include myxedema coma, cardiovascular disease, and pregnancy-related issues. Both hyperthyroidism and hypothyroidism can negatively impact a patient’s quality of life and psychological well-being; therefore, timely diagnosis and treatment are crucial (26, 27). Animal studies have found that caffeine may be associated with a decrease in TSH concentration (10), suggesting that coffee consumption could have endocrine-disrupting effects on thyroid function. L-thyroxine is a synthetic form of thyroid hormone (28). In various studies, coffee consumption has been shown to interfere with the absorption of L-thyroxine (29–33). In a study conducted by Marija Andjelkovic (34), which included 150 patients on thyroid replacement therapy with cardiovascular disease, they found that cigarette smoking was a risk factor that decreased TSH levels in patients on thyroid replacement therapy but did not find an effect of coffee consumption on patients’ TSH. An additional RCT included 11 healthy subjects, whose thyroid function levels were measured after receiving different types of coffee oil, and showed virtually no change in T4, T3, and TSH concentrations (35). However, these studies were influenced by the number of patients and the patients’ own underlying diseases, so large sample studies are needed to explore the effects of coffee consumption on thyroid function. Subclinical hypothyroidism affects up to 10% of adults, and patients with subclinical hypothyroidism are at significant risk of progressing to hypothyroidism (36). Additionally, subclinical hypothyroidism is associated with a variety of cardiovascular diseases and all-cause mortality (37, 38). Our study revealed that coffee consumption of <2 cups per day effectively reduced the risk of developing subclinical hypothyroidism (OR=0.60, 95% CI 0.41-0.88) (**Table 3**), a finding with some clinical significance. We speculate that the mechanism may be due to coffee consumption helping to control TSH concentration within the normal range of 0.45 mIU/- 4.5 mIU/L. Several studies exploring the effects of coffee consumption on humans using MR methods have been reported. Nordestgaard et al. (39) found that coffee consumption prevented symptomatic gallstone disease, while Kim et al. (40) showed that higher coffee consumption was associated with a lower risk of arrhythmias. These studies were analyzed based on MR methods. The results of this study, which analyzed data from subjects in NHANES, showed no effect of coffee consumption on the risk of hyperthyroidism and hypothyroidism. We then analyzed the causal relationship between coffee consumption and hyperthyroidism and hypothyroidism separately using the MR method, and the results remained non-significant, which validates our retrospective cross-sectional study and again supports the conclusion that coffee consumption is not associated with the risk of hyperthyroidism and hypothyroidism (**Table 4**). We also performed additional validation of pleiotropy and heterogeneity, as well as sensitivity analysis of the results using the leave-one-out method, to make the results more reliable. Nevertheless, this study has several limitations, such as the fact that compounds in coffee are affected by various factors, including the type of coffee, brewing method, and degree of coffee roasting. Additionally, we were unable to distinguish between caffeinated and decaffeinated coffee consumption. In this study, the source of data on coffee consumption was the same as the two 24-hour recall interviews in NHANES. Due to the retrospective nature of data collection, it does not accurately reflect individuals’ regular intake. To minimize bias, we excluded subjects who had only one 24-hour recall interview and averaged the data obtained from the two 24-hour recalls as coffee consumption. It has been suggested that two 24-hour recalls may be sufficient to assess daily dietary consumption (41). The data from the genome-wide association analysis used in our study were obtained from European populations, which limits the generalizability of our findings. Further research is needed among different ethnic groups to yield more comprehensive results.

In conclusion, our study has shed light on the relationship between coffee consumption and thyroid function, yet there are still aspects that warrant further exploration. To address the limitations of the current study and provide more robust evidence, large-scale, multi-ethnic, and prospective studies are required. As our understanding of the potential effects of coffee consumption on thyroid function grows, it will be crucial to consider various factors, such as the type of coffee, brewing method, and degree of coffee roasting, in future research.

## 5 Conclusions

Our study demonstrated that drinking <2 cups of coffee per day reduced the risk of subclinical hypothyroidism (OR=0.60, 95% CI: 0.41, 0.88) and 2-4 cups of coffee reduced serum TSH concentrations (b=-0.23, 95% CI: -0.30, -0.16) compared to no coffee consumption. In addition, coffee consumption was not associated with the risk of hyperthyroidism and hypothyroidism.

## Data availability statement

The original contributions presented in the study are included in the article/**Supplementary Material**. Further inquiries can be directed to the corresponding author.

## Ethics statement

The studies involving human participants were reviewed and approved by National Center for Health Statistics. The patients/participants provided their written informed consent to participate in this study.

## Author contributions

Conceptualization: GZ and RC. Methodology: GZ. Software: JJ. Validation: GZ, RC, and ZW. Formal analysis: GZ. Investigation: JJ. Resources: ZW. Data curation: RC. Writing—original draft preparation: GZ. Writing—review and editing: RC. Visualization: JJ. Supervision: RC. Project administration: RC. Funding acquisition: RC. All authors have read and agreed to the published version of the manuscript. All authors contributed to the article.
