# Supplementary material for: Effect of coffee consumption on thyroid function: NHANES 2007-2012 and Mendelian randomization
Source: Front Endocrinol (Lausanne). 2023 Jun 7;14:1188547. doi: 10.3389/fendo.2023.1188547 (PMC10282749; doi:10.3389/fendo.2023.1188547)

Supplementary Material

Article Title

**Guoxu Zhao, Zhao Wang, Jinli Ji, Rongjun Cui**

*** Correspondence:** Rongjun Cui:cuirongjun@mdjmu.edu.cn

# Supplementary Tables

Table S1: Multi-potency testing for MR analysis;

| IV | exposure | outcome | Egger_intercept | SE | P |
| --- | --- | --- | --- | --- | --- |
| IV_1 | Coffee | Hyperthyroidism | -0.000264676 | 0.000203933 | 0.2641177 |
|  | Coffee | Hypothyroidism | -0.000516105 | 0.000623381 | 0.4542555 |
| IV_2 | Coffee | Hyperthyroidism | -0.000217635 | 0.000348379 | 0.6445181 |
|  | Coffee | Hypothyroidism | -0.001785387 | 0.000852066 | 0.2834728 |

Table S2: MR analysis for heterogeneity detection;

| IV | exposure | outcome | method | Q | Q_pval |
| --- | --- | --- | --- | --- | --- |
| IV_1 | coffee | hyperthyroidism | MR Egger | 2.132607 | 0.711385 |
|  | coffee | hyperthyroidism | IVW | 3.817046 | 0.576046 |
|  | coffee | Hypothyroidism | MR Egger | 6.249736 | 0.181258 |
|  | coffee | Hypothyroidism | IVW | 7.320692 | 0.197862 |
| IV_2 | coffee | hyperthyroidism | MR Egger | 0.562662 | 0.45319 |
|  | coffee | hyperthyroidism | IVW | 0.952923 | 0.620977 |
|  | coffee | Hypothyroidism | MR Egger | 0.006477 | 0.935855 |
|  | coffee | Hypothyroidism | IVW | 4.397021 | 0.110968 |

Table S3: Details of instrumental variables in MR analysis.

| IV | Exposure | Outcome | SNP | effect_allele.exposure | other_allele.exposure | effect_allele.outcome | other_allele.outcome | beta.exposure | beta.outcome | se.exposure | se.outcome | pval.exposure | pval.outcome |
| --- | --- | --- | --- | --- | --- | --- | --- | --- | --- | --- | --- | --- | --- |
| IV_1 | Coffee | Hyperthyroidism | rs1260326 | T | C | T | C | -0.04 | 0.000172139 | 0.01 | 0.000184954 | 7.14E-08 | 0.35 |
|  | Coffee | Hyperthyroidism | rs1481012 | A | G | A | G | 0.06 | 0.000328397 | 0.01 | 0.000287024 | 8.93E-08 | 0.25 |
|  | Coffee | Hyperthyroidism | rs17685 | A | G | A | G | 0.07 | -5.65E-05 | 0.01 | 0.000201997 | 4.26E-11 | 0.780001 |
|  | Coffee | Hyperthyroidism | rs2472297 | T | C | T | C | 0.14 | 0.000373764 | 0.01 | 0.00020534 | 2.47E-24 | 0.0690001 |
|  | Coffee | Hyperthyroidism | rs4410790 | T | C | T | C | -0.1 | -6.87E-05 | 0.01 | 0.000187669 | 3.08E-17 | 0.709999 |
|  | Coffee | Hyperthyroidism | rs7800944 | T | C | T | C | -0.05 | -8.78E-06 | 0.01 | 0.000200719 | 2.29E-11 | 0.97 |
| IV_2 | Coffee | hyperthyroidism | rs17685 | A | G | A | G | 0.08 | -5.65E-05 | 0.01 | 0.000201997 | 1.13E-09 | 0.780001 |
|  | Coffee | Hyperthyroidism | rs2470893 | T | C | T | C | 0.2 | 0.000288202 | 0.02 | 0.000192224 | 5.05E-19 | 0.13 |
|  | Coffee | Hyperthyroidism | rs6968554 | A | G | A | G | -0.2 | -8.65E-05 | 0.03 | 0.000187959 | 7.41E-15 | 0.649999 |
| IV_1 | Coffee | Hypothyroidism | rs1260326 | T | C | T | C | -0.04 | 0.000175208 | 0.01 | 0.000452182 | 7.14E-08 | 0.7 |
|  | Coffee | Hypothyroidism | rs1481012 | A | G | A | G | 0.06 | 0.00111581 | 0.01 | 0.000701756 | 8.93E-08 | 0.11 |
|  | Coffee | Hypothyroidism | rs17685 | A | G | A | G | 0.07 | 0.000802716 | 0.01 | 0.000494041 | 4.26E-11 | 0.1 |
|  | Coffee | Hypothyroidism | rs2472297 | T | C | T | C | 0.14 | 0.000828988 | 0.01 | 0.000502324 | 2.47E-24 | 0.0990011 |
|  | Coffee | Hypothyroidism | rs4410790 | T | C | T | C | -0.1 | 0.000566423 | 0.01 | 0.000458999 | 3.08E-17 | 0.22 |
|  | Coffee | Hypothyroidism | rs7800944 | T | C | T | C | -0.05 | 0.000159261 | 0.01 | 0.000490916 | 2.29E-11 | 0.75 |
| IV_2 | Coffee | Hypothyroidism | rs17685 | A | G | A | G | 0.08 | 0.000802716 | 0.01 | 0.000494041 | 1.13E-09 | 0.1 |
|  | Coffee | Hypothyroidism | rs2470893 | T | C | T | C | 0.2 | 0.000698352 | 0.02 | 0.000470238 | 5.05E-19 | 0.14 |
|  | Coffee | Hypothyroidism | rs6968554 | A | G | A | G | -0.2 | 0.000645427 | 0.03 | 0.000459709 | 7.41E-15 | 0.16 |

# Supplementary Figure

Figure S1: Sensitivity testing of instrumental variables using the leave-one-out method;


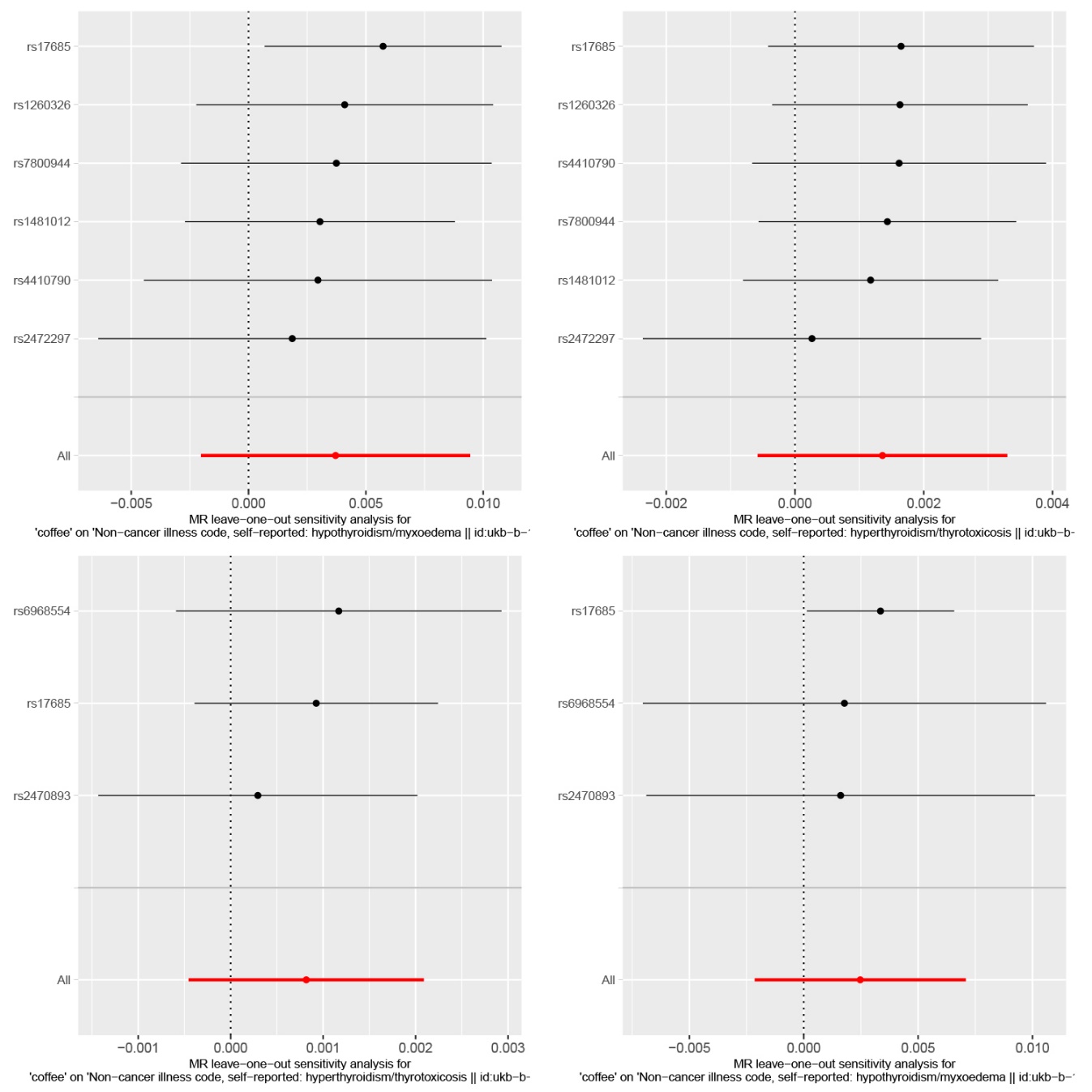

Supplement: Supplementary file 1 [file DataSheet_1.docx]
